# Bottom-Up (Cu, Ag, Au)/Al_2_O_3_/Bi_2_Te_3_ Assembled Thermoelectric Heterostructures

**DOI:** 10.3390/mi12050480

**Published:** 2021-04-22

**Authors:** Zhenhua Wu, Shuai Zhang, Zekun Liu, Cheng Lu, Zhiyu Hu

**Affiliations:** 1 National Key Laboratory of Science and Technology on Micro-Nano Fabrication, Shanghai Jiao Tong University, Shanghai 200240, China; wuzhenhua@sjtu.edu.cn (Z.W.); zhangs0521@sjtu.edu.cn (S.Z.); liuzekun@sjtu.edu.cn (Z.L.); lucheng_2020@sjtu.edu.cn (C.L.); 2 Department of Micro/Nano-Electronics, Shanghai Jiao Tong University, Shanghai 200240, China; 3 Institute of Nano-Micro Energy, Shanghai Jiao Tong University, Shanghai 200240, China

**Keywords:** thermoelectric, Bi_2_Te_3_, heterostructure, interface, metal, oxide

## Abstract

The interface affects the transmission behavior of electrons and phonons, which in turn determines the performance of thermoelectric materials. In this paper, metals (Cu, Ag, Au)/Al_2_O_3_/Bi_2_Te_3_ heterostructures have been fabricated from bottom to up to optimize the thermoelectric power factor. The introducing metals can be alloyed with Bi_2_Te_3_ or form interstitials or dopants to adjust the carrier concentration and mobility. In addition, the metal-semiconductor interface as well as the metal-insulator-semiconductor interface constructed by the introduced metal and Al_2_O_3_ would further participate in the regulation of the carrier transport process. By adjusting the metal and oxide layer, it is possible to realize the simultaneous optimization of electric conductivity and Seebeck coefficient. This work will enable the optimal and novel design of heterostructures for thermoelectric materials with further improved performance.

## 1. Introduction

Thermoelectric technology can convert heat to electricity freely. The thermoelectric (TE) efficiency is normally evaluated by the dimensionless figure of merit *ZT* = *S*^2^*σT*/*κ* (*κ* = *κ*_e_ + *κ*_L_), where *S*, *σ*, *κ*, *κ*_e_, *κ*_L_, and *T* are the Seebeck coefficient, electrical conductivity, thermal conductivity, electronic thermal conductivity, lattice thermal conductivity, and absolute temperature, respectively [1]. One strategy to improve the thermoelectric performance is fabricating nanostructures such as pores [2,3] or nanograins [4,5], which could strongly scatter phonons and thereby reducing the lattice thermal conductivity. However, little room remains for further reducing κ. On the one hand, the contribution of electrons to thermal conductivity is unavoidable due to Wiedemann-Franz law. On the other hand, the lattice thermal conductivity cannot be lower than the amorphous state. For Bi_2_Te_3_ system with excellent thermoelectric properties near room temperature, the lattice thermal conductivity has been approached to lower as ~0.3 W·m^−1^·K^−1^, which is the limit value predicted by Cahill model [6,7]. Further improvements in the *ZT* could be relied on decoupling *S* and *σ* to improve the power factor (*PF* = *S*^2^*σ*). 

Compared with its bulk counterparts, nanostructured Bi_2_Te_3_ film provides promising possibilities for enhanced TE properties and potential applications in micro/nano-electromechanical systems (MEMS/NEMS) TE device [8]. The thermoelectric performance of Bi_2_Te_3_ can be optimized by adjusting the (00*l*) orientation [9,10], pores [9,11], nanosheet boundary [12,13], and the intrinsic defects [14,15]. In addition, constructing a heterogeneous interface is also one of the effective methods to optimize the thermoelectric performance. The heterogeneous interface can filter low-energy carriers that contribute negative to the Seebeck coefficient through the interface barrier and thus the thermoelectric power factor will be enhanced [16]. Meanwhile, the interface could also scatter phonons to reduce the lattice thermal conductivity, and finally the *ZT* value of the material will be further improved [1]. Experimentally, metal/semi-metal or carbon had been introduced in Bi_2_Te_3_ to build heterostructures (such as Bi_2_Te_3_/Ag [12,17], Bi_2_Te_3_/Cu [12,18] Bi_2_Te_3_/Cd [19], Bi_2_Te_3_/Te [20], Bi_2_Te_3_/carbon nanotube [10,21]) especially by solution or sintering method to further enhanced the TE properties. Oxide layer was also introduced into the Ag/Sb_2_Te_3_ system by atomic layer deposition, and the filtering effect of the corresponding interface on low-energy carriers was discussed [6]. In this paper, group IB metals (M = Cu, Ag, Au) and Al_2_O_3_ interfaces were systematically introduced into Bi_2_Te_3_ film to modulate the carrier behavior and thus enhance the thermoelectric power factor. Incidentally, the introduced interface can also scatter phonons to reduce the thermal conductivity of the lattice, which is beneficial to improve the *ZT* value.

## 2. Materials and Methods

**Figure 1:**
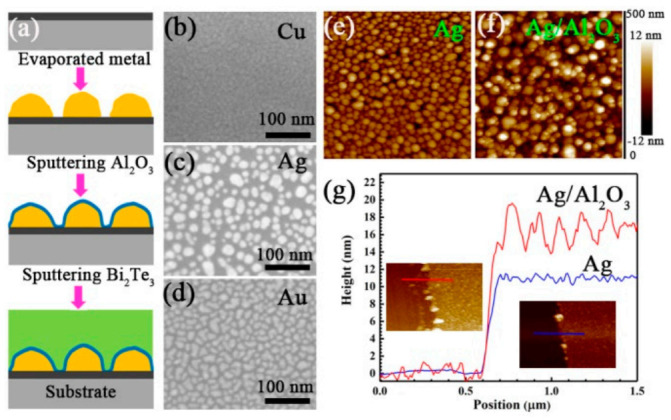
(**a**) Film preparation process. SEM image of (**b**) Cu, (**c**) Ag and (**d**) Au surface. AFM image of (**e**) Ag and (**f**) Ag/ Al_2_O_3_. (**g**) AFM step profiler of Ag and Ag/Al_2_O_3_.

Group IB metal M (M = Cu, Ag, Au) with a thickness of 6~10 nm was pre-deposited on SiO_2_/Si substrates by an e-beam evaporation system. Using Al_2_O_3_ targets, Al_2_O_3_ layer with a thickness of ~3 nm was first selectively sputtered on the pre-deposited metal. Then, Bi_2_Te_3_ films with a thickness of about 300 nm were sputtered. The base vacuum of the sputtering chamber was 5E^−3^ Pa and the working pressure was 0.26 Pa. The substrate was rotated at a speed of 20 rpm to ensure the uniformity of the sputtered film. The Al_2_O_3_ sputtered at 100 W and Bi_2_Te_3_ sputtered at 50 W were both in a radio frequency (RF) mode. The purity of all source materials is 99.99%. The sample preparation is illustrated in Figure 1a. For the convenience of description in the following part, pure Bi_2_Te_3_ is marked as B. Cu/Bi_2_Te_3_, Ag/Bi_2_Te_3_, and Au/Bi_2_Te_3_ samples are marked as CB, SB, and GB, respectively. Cu/Al_2_O_3_/ Bi_2_Te_3_, Ag/Al_2_O_3_/Bi_2_Te_3_, and Au/Al_2_O_3_/ Bi_2_Te_3_ are marked as CAB, SAB, and GAB, respectively.

The surface morphology, cross section, composition, and thickness of films were characterized by atomic force microscope (AFM, Multimode Nanoscope IIIa), scanning electron microscopy with energy dispersive spectrometer (SEM-EDS, Zeiss Ultra Plus) and surface profiler (KLA-Tencor P7). The structures of the films were characterized by X-ray diffraction (XRD, D8 Advance) with a Cu Kα radiation (λ = 1.5406Å) in conventional θ–2θ mode from 10–80° at a sweep rate of 4°/min. Electrical transport of film was obtained by Hall measurement (model: MMR) with a Van der Pauw pattern and averaged by three measurements. The contact effect and other additional effect including heating effect of current and asymmetric effect of probing electrodes can be exclude in this current reversal method with four-probe configuration. The Seebeck coefficient was evaluated by a home-built system as reported in detail in our previous study [22].

## 3. Results and Discussion

### 3.1. Heterostructure Characterization

The pre-deposited metal presents as discontinuous particles as shown in Figure 1b–d. The height of Ag is about 10 nm, while the height of Cu and Au is about 6–7 nm as shown in Figure 1 and Figure S1. After sputtering Al_2_O_3_, the total height of the metal particles increased by ~3 nm. Interestingly, Al_2_O_3_ appeared as smaller particles on the surface of the pre-deposited metal particles, resulting in increased particle fluctuations, as shown in Figure 1e–g.

**Figure 2:**
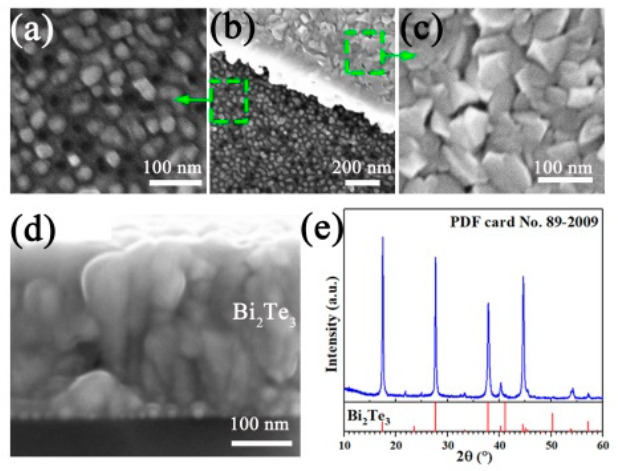
SEM image of (**a**) Ag/Al_2_O_3_, (**b**) Ag/Al_2_O_3_/Bi_2_Te_3_, and (**c**) Bi_2_Te_3_. (**d**) Cross section of Ag/Al_2_O_3_/Bi_2_Te_3_. (**e**) XRD pattern of Bi_2_Te_3_.

The Te/Bi atomic ratio of all Bi_2_Te_3_ films that sputtered with the same target and sputtering power was about ~1.5. The surface and cross section of Bi_2_Te_3_ and M/Al_2_O_3_/Bi_2_Te_3_ are given in Figure S2. Take Ag/Al_2_O_3_/Bi_2_Te_3_ as an example, the surface of each layer is given in Figure 2a–c. Regardless of whether metal/oxide was previously deposited, there was no obvious difference in the surface morphology of the Bi_2_Te_3_ film, the surface of which is selected as shown in Figure 2c. The bottom-up Ag/Al_2_O_3_/Bi_2_Te_3_ structure could be clearly seen from the cross section as shown in Figure 2d. The thickness of the Bi_2_Te_3_ film was about 300 nm, and the XRD pattern of Bi_2_Te_3_ indicated that the obtained Bi_2_Te_3_ is with good crystallinity as shown in Figure 2e. 

### 3.2. Carrier Transport Characteristics

**Figure 3:**
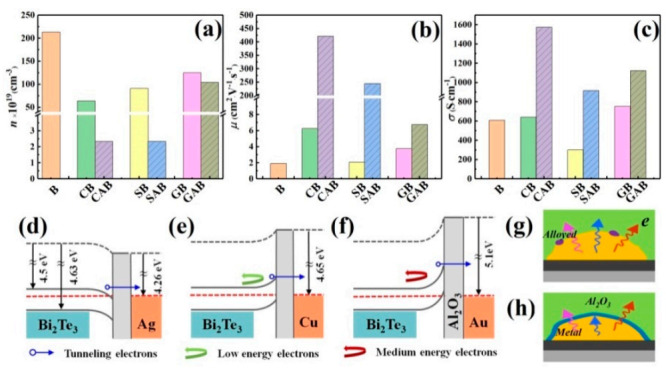
Electrical properties of Bi_2_Te_3_-based films at 300 K. (**a**) Carrier concentration (*n*). (**b**) Carrier mobility (*μ*). (**c**) Electrical conductivity (*σ*). Equilibrium band gap alignment of (**d**) Ag, Al_2_O_3_ and Bi_2_Te_3_, (**e**) Cu, Al_2_O_3_, and Bi_2_Te_3_, (**f**) Au, Al_2_O_3_, and Bi_2_Te_3_. Behavior of electrons at the interface of (**g**) Metal/Bi_2_Te_3_ and (**h**) Metal/Al_2_O_3_/Bi_2_Te_3_.

All Bi_2_Te_3_-based films exhibit n-type conductivity, and the majority of carriers in the films are electrons as characterized by Hall measurements and shown in Figure 3a–c. Compared with the pure Bi_2_Te_3_ film, the introduction of M (M = Cu, Ag, Au) in the film leads to a decrease in total electron concentration of the film as shown in Figure 3a.

The role of introducing group IB metals in B_i2_Te_3_ can be summarized as follows: (i) In one way, one M atom should produce one electron compensating a hole. This process can be achieved by forming [MBiTe_2_ +Bi_3_Te_4_^−^]^−^ through the alloying of Bi_2_Te_3_ and M as shown in Figure 3g [23]. Generally, the diffusion, solid solution and alloying process of group IB metal in Bi_2_Te_3_ can be modulated by heat treatment [23,24,25,26]. Because the films in this study have not been annealed, the above effects, especially the alloying process (generate new phases), are weak. (ii) Another way is that the introduction of M atom could form interstitial M_i_^+^ compensated by electrons. While the electronegativity (Pauling scale) of Cu, Ag, and Au atoms increases in turn (from 1.90, 1.93 to 2.54), the ability to form M_i_^+^ decreases in turn [14]. Generally, since the space in the lattice is limited, atoms or cations with a smaller radius are more likely to form interstitial phases. The radius of Cu, Ag, and Au are 1.28 Å, 1.44 Å, and 1.44 Å, respectively. The radius of Cu^+^, Ag^+^, and Au^+^ are 0.98 Å, 1.10 Å, and 1.10 Å, respectively [27]. In terms of the ability to form interstitial, the electron concentration of the Cu-introduced film should be less than that in the Ag-introduced film, which is confirmed in Figure 3a. (iii) Since M (M = Cu, Ag, and Au) have fewer electrons in their outermost orbitals than Bi, doping them at the Sb-sites greatly increases the hole concentration and consequently reduces the electron concentration [19].

The coexistence of the above alloying, interstitial and doping process together determines the reduction of the electron concentration in the Bi_2_Te_3_ film after the introduction of metal. Eventually, the electron concentration of the Bi_2_Te_3_, Au/Bi_2_Te_3_, Ag/Bi_2_Te_3_, and Cu/Bi_2_Te_3_ film decreases sequentially. After the Al_2_O_3_ layer was deposited to build a metal-insulator-semiconductor (MIS) interface, the electrons in the metal were blocked from entering Bi_2_Te_3_ and some carriers may tunnel through the thin oxide layer from the metal into Bi_2_Te_3_ as shown in Figure 3g,h. Because there were unavoidable narrow holes in such thin Al_2_O_3_ layer deposited by magnetron sputtering, there would still be some interfaces where the metal was in direct contact with Bi_2_Te_3_. The above-mentioned combined effect made the carrier concentration of the film further decrease after the Al_2_O_3_ layer was introduced.

As the electron concentration decreased after metal or oxide was introduced, the scattering between electrons was weakened and the mobility of the film increased. On the other hand, the introduction of a metal-semiconductor (MS) interface or an MIS interface would lead to the reduction of the mobility which was closely related to the size and distribution of the metal particles and the surface Al_2_O_3_ that covers them. The interfaces and the corresponding electronic transport processes are illustrated in Figure 3d–f. The above combined effect makes the total electrical mobility and conductivity of the films other than Ag/Bi_2_Te_3_ increase while compared to the pure Bi_2_Te_3_, which may be related to the Ohmic contact of the Ag-Bi_2_Te_3_ interface and Ag particle with relatively high height (thickness). In addition to filtering out low-energy electrons based on the interface barrier, an additional oxide layer was deposited to control the tunneling electron concentration. Through the above dual effects, the concentration and average energy of electrons can be adjusted.

### 3.3. Thermoelectric Properties

**Figure 4:**
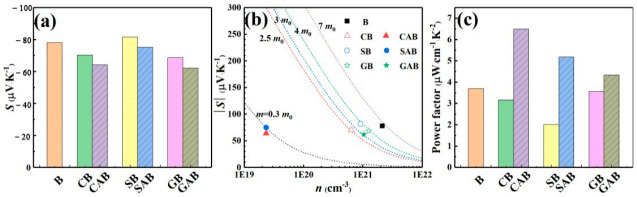
Thermoelectric properties of Bi_2_Te_3_-based films at 300 K. (**a**) Seebeck coefficient (*S*). (**b**) Seebeck coefficient as a function of carrier concentration (Pisarenko plot). Dashed lines represent theoretical fits for m* = 0.3 *m*_0_, 2.5 *m*_0_, 3 *m*_0_, 4 *m*_0_ and 7 *m*_0_ based on Equations (1)–(3). *m*_0_ is mass of free electron. (**c**) Power factor (*S*^2^*σ*).

The Seebeck coefficient is closely related to the carrier concentration and its energy distribution, and the contribution of low-energy carriers to the Seebeck coefficient is negative [28]. The measured Seebeck coefficient of Bi_2_Te_3_-based film is presented in Figure 4a. It can be seen from Figure 4a that the Seebeck coefficients of the film with Cu and Au are lower than the pure Bi_2_Te_3_, which may be related to the relatively high interface barrier that filters out some high-energy electrons. The deposited Al_2_O_3_ layer allowed tunneling electrons to pass through, which would reduce the concentration of high-energy electrons in the film, and ultimately reduce the Seebeck coefficient.

For a material with a single parabolic band, the Seebeck coefficient, DOS effective mass of carriers (m*) and Lorenz number (*L*) can be written as [22,29]

(1) $$S=\frac{K_{B}}{e}(\frac{\left(5/2+\lambda )F_{3/2+\lambda }(\eta \right)}{\left(3/2+\lambda )F_{1/2+\lambda }(\eta \right)}-\eta )$$

(2) $$m^{*}=\frac{h^{2}}{2K_{b}T}{\left(\frac{n}{4\pi F_{1/2}(\eta )}\right)}^{2/3}$$

(3) $$F_{i}(\eta )=\int_{0}^{\infty }\frac{x^{i}dx}{1+\exp(x-\eta )}$$

(4) $$L={\left(\frac{k_{B}}{e}\right)}^{2}\left\{\frac{\left(\lambda +7/2)F_{\lambda +5/2}(\eta \right)}{\left(\lambda +7/2)F_{\lambda +1/2}(\eta \right)}-{\frac{\left(\lambda +5/2)F_{\lambda +3/2}(\eta \right)}{\left(\lambda +3/2)F_{\lambda +1/2}(\eta \right)}}^{2}\right\}$$

*κ*_e_ = *LTσ* (5)

where *e*, m*, *h*, *k*_B,_ *L*, and *T* are the electronic charge, effective mass, Planck’s constant, Boltzmann constant, Lorenz number, and absolute temperature, respectively. *η* is the reduced Fermi energy (*E_F_*/*k_B_T*), *F_i_* is the fermi integral of the order of x. Acoustic phonon scattering (*γ* = −1/2) has been assumed as the main carrier scattering mechanism at room temperature. The corresponding calculation results at 300 K are listed in Table 1. In order to compare the effect of interface on the Seebeck coefficient of Bi_2_Te_3_ at room temperature, Pisarenko plot is applied as shown in Figure 4b. 

After the metal was introduced into Bi_2_Te_3_ film, the m* of the film was reduced, and it was further reduced after the oxide layer was introduced as shown in Table 1. However, when compared with Cu-introduced and Ag-introduced films, the effective mass change in the Au-introduced film was small, which was closely related to the carrier behavior as analyzed before. The power factor of Bi_2_Te_3_-based film at 300 K is given in Figure 4c. Compared with pure Bi_2_Te_3_, the power factor of the film introduced with metal and oxide is increased, which benefits from the effect of metals and the MIS interface. As a result, the power factor of the Cu/Al_2_O_3_/Bi_2_Te_3_ film is 6.5 μW·cm^−1^·K^−2^, which is 1.75 times that of a single Bi_2_Te_3_ film. In addition, the corresponding electronic thermal conductivity calculated according to Equation (5) is given in Table 1.

Although the introduction of Cu/Al_2_O_3_ leads to an increase in the electronic thermal conductivity of the film, the smaller Cu particles result in a higher interface density (Figure 1b), which could scatter phonons more strongly and make the lattice thermal conductivity lower [30]. In addition to scattering phonons at the interface to reduce the thermal conductivity, it is particularly important to use the interface to optimize the thermoelectric power factor to improve the overall performance of the film. 

## 4. Conclusions

In summary, metal (Cu, Ag, Au)/Al_2_O_3_/Bi_2_Te_3_ heterostructures have been fabricated to improve the thermoelectric properties. By constructing the metal-semiconductor and metal-insulator-semiconductor interface, using the filter carrier effect or controlling the tunneling electrons, it is possible to coordinately control the carrier concentration and mobility, and finally optimize the thermoelectric power factor. The power factor of the Cu/Al_2_O_3_/Bi_2_Te_3_ heterostructure film is 6.5 μW·cm^−1^·K^−2^, which is 1.75 times that of a single Bi_2_Te_3_ film. Future work will focus on optimizing the metal particle size, oxide layer thickness, and annealing process to further optimize the thermoelectric performance. Related works are still ongoing.

## Figures and Tables

**Table 1:** Thermoelectric parameters of Bi_2_Te_3_-based films at 300 K.

Sample	B	CB	CAB	SB	SAB	GB	GAB
m*/*m*_0_	6.38	2.56	0.26	3.80	0.30	3.92	3.12
*L* (V^2^·K^−2^)	2.04 × 10^−8^	2.08 × 10^−8^	2.12 × 10^−8^	2.01 × 10^−8^	2.05 × 10^−8^	2.09 × 10^−8^	2.14 × 10^−8^
*κ*_e_ (W·m^−1^·K^−1^)	0.37	0.40	1.00	0.18	0.56	0.47	0.72

## Supplementary Materials

The following are available online at https://www.mdpi.com/article/10.3390/mi12050480/s1, Figure S1: AFM step profiler of (**a**) Cu and (**d**) Au. Figure S2: Surface of (**a**) Bi_2_Te_3_, (**b**) Cu/Al_2_O_3_/Bi_2_Te_3_, (**c**) Ag/Al_2_O_3_/Bi_2_Te_3_, (**d**) Au/Al_2_O_3_/Bi_2_Te_3_. Cross section of (**e**) Bi_2_Te_3_, (**f**) Cu/Al_2_O_3_/Bi_2_Te_3_, (**g**) Ag/Al_2_O_3_/Bi_2_Te_3_, (**h**) Au/Al_2_O_3_/Bi_2_Te_3_.
