# Supplementary material for: Bottom-Up (Cu, Ag, Au)/Al2O3/Bi2Te3 Assembled Thermoelectric Heterostructures
Source: Micromachines (Basel). 2021 Apr 22;12(5):480. doi: 10.3390/mi12050480 (PMC8145968; doi:10.3390/mi12050480)
Supplement: Supplementary file 1 [file micromachines-12-00480-s001.zip › micromachines-1181797-supplementary.pdf]

micromachines

Article

Bottom-Up (Cu, Ag, Au)/Al_2O_3/Bi_2Te_3 Assembled Thermoelectric Heterostructures

Zhenhua Wu [1,2,3], Shuai Zhang [1,2,3], Zekun Liu [1,2,3], Cheng Lu [1,2,3] and Zhiyu Hu [1,*]

1 National Key Laboratory of Science and Technology on Micro-Nano Fabrication, Shanghai Jiao Tong University, Shanghai 200240, China; wuzhenhua@sjtu.edu.cn (Z.W.); zhangs0521@sjtu.edu.cn (S.Z.); liuzekun@sjtu.edu.cn (Z.L.); lucheng_2020@sjtu.edu.cn (C.L.)
2 Department of Micro/Nano-Electronics, Shanghai Jiao Tong University, Shanghai,200240,China
3 Institute of Nano-Micro Energy, Shanghai Jiao Tong University, Shanghai 200240, China
* Correspondence: zhiyuhu@sjtu.edu.cn

Citation: Wu, Z.; Zhang, S.; Liu, Z.; Lu, C.; Hu, Z. Bottom-Up (Cu, Ag, Au)/Al_2O_3/Bi_2Te_3 Assembled Thermoelectric Heterostructures. *Micromachines* **2021**, *12*, x, https://doi.org/10.3390/mi12050480

Academic Editors: Anton Köck and Marco Deluca

Received: 29 March 2021
Accepted: 19 April 2021
Published: 22 April 2021

Publisher's Note: MDPI stays neutral with regard to jurisdictional claims in published maps and institutional affiliations.

Copyright: © 2021 by the authors. Submitted for possible open access publication under the terms and conditions of the Creative Commons Attribution (CC BY) license (http://creativecommons.org/licenses/by/4.0/).

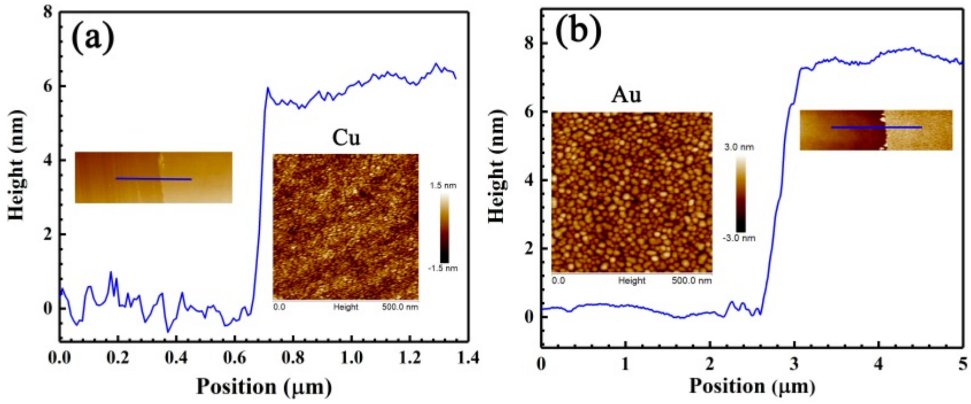

Figure S1 AFM step profiler of (a) Cu and (d) Au.

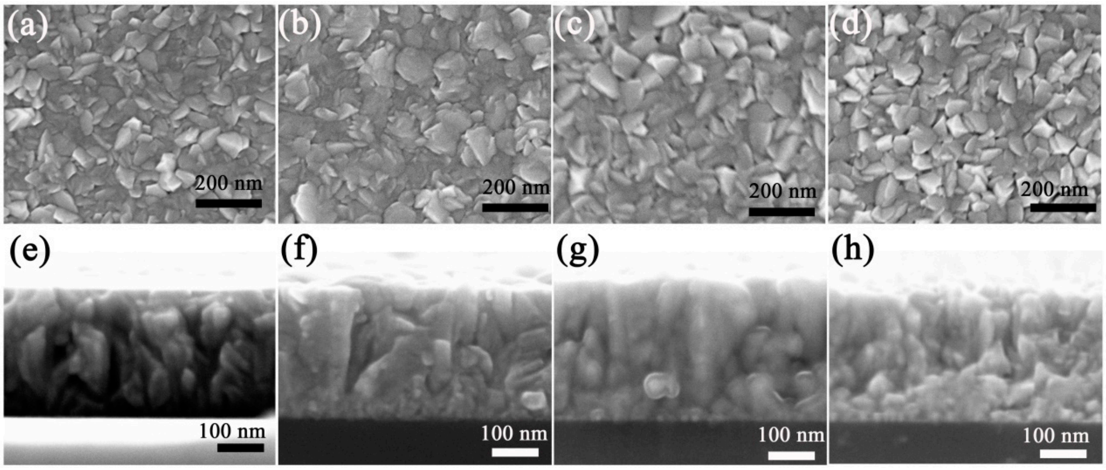

Figure S2 Surface of (a) Bi_2Te_3, (b) Cu/Al_2O_3/Bi_2Te_3, (c) Ag/Al_2O_3/Bi_2Te_3, (d) Au/Al_2O_3/Bi_2Te_3. Cross section of (e) Bi_2Te_3, (f) Cu/Al_2O_3/Bi_2Te_3, (g) Ag/Al_2O_3/Bi_2Te_3, (h) Au/Al_2O_3/Bi_2Te_3.
